# Dynamic cardiac dyssynchrony is strongly associated with 2-year dialysis adequacy in continuous ambulatory peritoneal dialysis patients

**DOI:** 10.1186/1471-2369-14-68

**Published:** 2013-03-23

**Authors:** Ching-Hui Huang, Chia-Chu Chang, Tzu-Lan Chang, Yu-Jun Chang

**Affiliations:** 1 Division of Cardiology, Department of Internal Medicine, Changhua Christian Hospital, Changhua, Taiwan; 2 Vascular and Genomic Research Center, Changhua Christian Hospital, Changhua, Taiwan; 3 Division of Nephrology, Department of Internal Medicine, Changhua Christian Hospital, Changhua, Taiwan; 4 Epidemiology and Biostatistics Center, Changhua Christian Hospital, Changhua, Taiwan; 5 School of Medicine, Chung Shan Medical University, Taichung, Taiwan

**Keywords:** 3D dobutamine echocardiography, Continuous ambulatory peritoneal dialysis, Left ventricular dyssynchrony

## Abstract

**Background:**

Left ventricular (LV) dyssynchrony is associated with increased risk of all-cause mortality in patients with end-stage renal disease. Our aim was to determine the associations of LV dynamic dyssynchrony with peritoneal solute clearance in continuous ambulatory peritoneal dialysis (CAPD) patients. Our primary objective was to determine the association between dynamic LV dyssynchrony and CAPD clearance at 2 years. Secondary objectives were to identify the factors influencing dynamic dyssynchrony, and to examine the association between dialysis adequacy and echocardiography-assessed LV outcomes.

**Methods:**

Fifty CAPD patients and 13 healthy volunteers underwent three-dimensional (3D) dobutamine stress echocardiography (DSE). The main endpoint was systolic dyssynchrony index (SDI). Secondary endpoints, including NT-proBNP, troponin I, Kt/V, and biochemical parameters, were measured before stress echocardiography, and Kt/V was measured again 2 years later. All values are expressed as medians and interquartile ranges (IQR).

**Results:**

NT-proBNP (3872 [808–11779] vs. 4.99 [4.99–36.83] pg/mL, *P* < 0.001), and log NT-proBNP (3.587 [2.896–4.071] vs. 0.698 [0.698–1.540], *P* < 0.001) levels were significantly higher in the CAPD group than in the control group. Real-time 3D DSE showed that the systolic dyssynchrony index was significantly different between the two groups at the peak dobutamine stage (1.11% [0.76–1.64%] vs. 0.66% [0.50–1.02%], *P* = 0.004), but not at resting (1.30% [0.89–1.74%] vs. 1.22 % [0.72–1.68%], *P* = 0.358).The subgroup of patients in the CAPD group with greater improvements in dialysis adequacy had lower baseline dynamic SDI and more favorable echocardiographic findings at 2 years. Dialysis adequacy decreased significantly at 2 year in patients with higher, but not in those with lower dynamic SDI at baseline. In multivariate linear regression analysis, log NT-proBNP and SDI at the peak dobutamine dose were significantly associated with Kt/V and weekly creatinine clearance at 2 years, while log NT-proBNP was significant associated with SDI at the peak dobutamine stage. Female CAPD patients group had more pronounced dynamic LV dyssynchrony compared with male patients.

**Conclusions:**

Dynamic systolic dyssynchrony was strongly associated with future dialysis adequacy in CAPD patients. Log NT-proBNP was the important predictor of dynamic dyssynchrony. Our study confirmed the concept that cardiac dysfunction has an impact on dialysis adequacy.

## Background

Solutes are transported from peritoneal capillaries to the dialysate by diffusion and convection processes. Diffusion is the most important transport mechanism for low molecular weights solutes [1]. Recent studies have demonstrated that transperitoneal clearance of small solutes is limited by the rate of blood flow during peritoneal dialysis [2]. The local renin–angiotensin–aldosterone system (RAAS), which regulates cardiac hemodynamic status and hypertension, plays an important role in the fibrotic process [3].

Therefore, we hypothesized that cardiac dysfunction is associated with small solute clearance in continuous ambulatory peritoneal dialysis (CAPD) patients.

Left ventricular (LV) compliance is an important determinant of LV function and can be affected by a variety of cardiovascular conditions. Diastolic dysfunction is also associated with altered LV compliance [4]. Transthoracic three-dimensional echocardiography (3DE) confirmed the feasibility of noninvasive estimation of the end-diastolic pressure–volume relationship. 3DE was also able to differentiate normal from abnormal LV compliance, which may be difficult or impossible to do using conventional 2DE [5].

N-terminal prohormone of brain natriuretic peptide (NT-proBNP) levels are correlated with LV wall stress, sphericity index (SI), and the extent of myocardial damage in patients with coronary artery disease (CAD), and with volume overload in heart failure. NT-proBNP is also an independent predictor of mortality in patients with ESRD [6-8]. However, no studies have examined whether NT-ProBNP can serve as a surrogate marker for dynamic LV dyssynchrony.

From this context, the main objective of this study was to investigate the association between LV dynamic dyssynchrony and CAPD clearance 2 years later. Our secondary objectives were to identify which factors influence dynamic dyssynchrony and to determine the association between dialysis adequacy and echocardiography-determined LV outcomes.

## Methods

### Subjects

Fifty uremic subjects on CAPD and 13 healthy volunteers were recruited from Changhua Christian Hospital, Changhua, Taiwan, between 2008 and 2011. All of the subjects were aged > 18 years and had normal sinus rhythm. None of the subjects in the control group were taking any medications. Subjects with acute coronary syndrome, a history of myocardial infarction, acute heart failure, congenital heart disease, severe valvular heart disease, or severe uncontrolled hypertension (systolic blood pressure [SBP] ≥ 210 mmHg or diastolic blood pressure [DBP] ≥ 110 mmHg) were excluded. Subjects with recent (< 3 months) peritonitis, other technique failure, or recent hospitalization were also excluded.

All CAPD patients received 2 L of 5% glucose peritoneal dialysate (Dianeal®; Baxter, Singapore) during transthoracic 3DE. The calculated peritoneal creatinine clearance rate was normalized to 1.73 m^2^ of the body surface area, and residual function was also taken into account. Membrane transport status was analyzed using a standard 4-h peritoneal equilibration test (PET). Dialysis adequacy was calculated as Kt/V, where K = dialyzer clearance of urea, t = dialysis time, and V = volume distribution of urea.

The protocol was approved by Institutional Review Board and the Ethics Committee of the Changhua Christian Hospital, Taiwan. All enrolled subjects provided written informed consent.

### Dobutamine stress echocardiography (DSE)

Subjects underwent a standard dobutamine stress protocol, under continuous electrocardiography monitoring, with infusions of 5, 10, 20, 30, and 40 μg/kg/min dobutamine for 3 min at each stage. Heart rate and BP were recorded at baseline, during each stage, and during recovery (3 min after termination of infusion). Endpoints for study termination were extensive new wall motion abnormalities, attainment of the target heart rate (85% of the maximal heart rate predicted for age), or completion of the protocol. Images were acquired at baseline, during the final minute of each stage, and during recovery. All images were acquired by the same cardiologist.

### Real-time (RT)3DE

RT3DE images were obtained with an IE33 xMATRIX echocardiography system (Philips Medical Systems, Andover, MA) and an X3-1 transducer (2–4 MHz) using secondary harmonic imaging. RT3DE images were acquired from the apical window immediately after the acquisition of 2DE images at each stage using another IE33 machine. Full-volume LV datasets were obtained for offline analysis using a QLab system (Philip Medical Systems). LV datasets were divided into 16 segments, excluding the apex, as recommended by the American Society of Echocadiography [9].

We used the dyssynchrony index, as previously described by Kapetanakis et al [10]. The systolic dyssynchrony index (SDI) was defined as the standard deviation of the time-to-minimum systolic volume of 16 LV segments expressed as a percentage of the R–R duration.

### 2DE

All 2DE parameters were measured using techniques recommended by the American Society of Echocadiography [9]. Systolic wall stress before dobutamine stress echocardiography (DSE) was calculated according to the formula of Grossman et al [11]. Left atrial (LA) volume was calculated using the biplane area–length method using 4- and 3-chamber views [4].

### Measurement of NT-proBNP and troponin I levels

Fasting (≥8 h) venous blood samples (4 mL) were obtained before starting DSE. NT-proBNP was measured using an immunoassay (ECLIA; Roche Diagnostics GmbH, Mannheim, Germany), and troponin I was measured using a chemiluminescent immunoassay (Access AccuTnI assay; Beckman Coulter Inc., Brea, CA). NT-proBNP levels were logarithmically transformed before statistical analyses because the values were not normally distributed.

### Statistical analysis

All statistical procedures were performed using SPSS software version 15.0 (SPSS Inc., Chicago, IL). All values are expressed as medians (interquartile range, IQR) Intergroup differences were determined using the Mann–Whitney test and *P* values < 0.05 were considered to be statistically significant. Intragroup differences (i.e., baseline vs. 2 years) were evaluated using the Mann–Whitney test and Wilcoxon test. Spearman’s ρ correlation and multivariate linear regression analyses were used to determine the associations between echocardiographic parameters and dialysis adequacy. Parameters showing significant correlations in univariate analyses and those with clinical relevance were then included in stepwise multiple linear regression analysis.

### Intraobserver and interobserver variability

Intraobserver variability was determined by repeating the offline 3DE measurements of SDI at 2 weeks after baseline in all subjects. Interobserver variability was determined by comparison of all offline measurements by two cardiologists who were blinded to each other’s interpretations. Variability values were calculated as the absolute difference between the corresponding measurements in terms of the mean percentage.

## Results

### Subject characteristics

Table 1 summarizes the characteristics of CAPD patients and the healthy volunteers. Both groups were matched for BMI, age, and sex. NT-proBNP (3872 [808–11779] vs.4.99 [4.99–36.83] pg/mL, *P* < 0.001), log NT-proBNP (3.587 [2.896–4.071] vs. 0.698 [0.698–1.540] *P* < 0.001), and systolic wall stress (61.7 [43.11–100.0] vs. 40.5 [29.6–48.6] mmHg, *P* = 0.001) values were significantly higher in the CAPD group than control group. RT3DE showed evidence of dynamic systolic dyssynchrony in the CAPD group. SDI was significantly different between the two groups, but only at the peak dobutamine dose (1.11% [0.76–1.64%] vs. 0.66% [0.50–1.02%], *P* = 0.004).

**Table 1:** Subject characteristics

	**Control group**		**CAPD group**		***P***
*n*		13		50	
Age, y	43.0	(32.0–52.0)	37.0	(35.0–44.3)	0.189
Male/female (*n*)	7/6		28/22		0.193
BSA (kg/m^2^)	1.71	(1.52–1.80)	1.68	(1.43–1.95)	0.609
Height (cm)	161.0	(155.5–167.5)	164.0	(153.0–168.3)	0.474
Weight (kg)	62.0	(52.0–72.3)	64.0	(47.5–79.0)	0.878
NT-proBNP (pg/mL)	4.99	(4.99–36.83)	3872	(808–11779)	<0.001
Log NT-proBNP	0.698	(0.698–1.540)	3.587	(2.896–4.071)	<0.001
SDI at baseline (%)	1.22	(0.72–1.68)	1.30	(0.89–1.74)	0.358
SDI at peak DSE (%)	0.66	(0.50–1.02)	1.11	(0.76–1.64)	0.004
SWS (mmHg)	40.5	(29.6–48.6)	61.7	(43.11–100.0)	0.001

Values are medians (interquartile range). *BSA*, body surface area; *CAPD*, continuous ambulatory peritoneal dialysis; *NT-proBNP*, N-terminal pro-B-type natriuretic peptide; *SWS*, systolic wall stress.

### Evaluation of SDI at the peak stage of DSE and 2-year Kt/V in CAPD patients

One subject who underwent kidney transplantation and one subject who switched to hemodialysis were excluded from the follow-up study. The other 48 subjects in the CAPD group underwent follow-up 2DE and resting 3DE 2 years after the original examinations. Positive values for 2-year Kt/V – baseline Kt/V (ΔKt/V) indicated better clearance function. Patients with a positive ΔKt/V value had lower values of the LV mass index (MI), LV end-diastolic volume (EDV) index, and LV end-systolic volume (ESV) index, as well as lower SDI at the peak stage of DSE, as compared with patients with a negative ΔKt/V value (Table 2). The median SDI in the CAPD group at the peak dobutamine dose was 1.1%. Using this as a reference value, CAPD patients were divided into two subgroups according to SDI at the peak dobutamine dose as having high (i.e., >1.1%) or low (≤1.1%) dynamic SDI. The data are shown in Table 3. Dialysis adequacy at 2 years had decreased significantly from baseline in the high SDI subgroup, but not in the low SDI subgroup. We found no significant difference in troponin I concentrations between patients with low or high SDI (0.025 [0.09–0.05] vs. 0.035 [0.009–0.05] mg/mL, *P* = 0.247).

**Table 2:** Kt/V and echocardiographic parameters at baseline and at 2 years

	**Patients with an improvement in Kt/V**		**Patients with a worsening in Kt/V**		***P***
*n*	14		34		
**Baseline**					
LVM (g)	82.2	(77.6–91.3)	188.8	(107.8–201.9)	<0.001
LVMI (g/m^2^)	61.8	(51.0–63.4)	95.3	(76.4–107.9)	<0.001
LVIDd (mm)	40.0	(34–44)	56.0	(47–60)	<0.001
LVIDs (mm)	25.0	(23.0–26.0)	39.0	(27.5–43.0)	0.004
LA (mm)	32.0	(25.0–39.0)	40.0	(33.5–45.0)	0.012
RV (mm)	19.0	(15.0–20.0)	22.0	(20.3–25.3)	0.002
IVS (mm)	9.0	(7.0–12.0)	12.0	(10.5–16.0)	0.002
LVPW (mm)	9.0	(8.0–9.0)	11.5	(8.8–13.0)	0.014
SWS	44.22	(33.87–63.26)	61.680	(52.00–100.02)	0.027
EDV index (ml/m^2^)	27.30	(24.14–39.10)	50.05	(44.70–56.30)	<0.001
ESV index (ml/m^2^)	8.8	(4.93–14.51)	18.00	(12.38–22.50)	<0.001
LA volume (ml)	34.6	(18.22–41.5)	68.0	(35.1–70.1)	0.002
LA volume index (ml/m^2^)	22.76	(13.70–28.83)	34.39	(25.78–38.10)	0.002
SDI at peak DSE (%)	0.71	(0.61–0.86)	1.51	(1.11–2.24)	<0.001
SDI at baseline (%)	1.20	(0.95–1.57)	1.35	(0.78–3.13)	0.688
EF (%)	64.0	(62.9–82.0)	62.7	(57.6–74.7)	0.056
logNT-proBNP	2.96	(2.70–3.30)	3.9	(3.23–4.50)	0.002
NT-proBNP	904.0	(502.0–1996.0)	7494.0	(2025.8–31357.0)	0.002
Kt/V	2.00	(1.96–2.06)	2.08	(1.79–2.33)	0.365
WCC	51.35	(44.93–79.83)	67.63	(61.13–79.4)	0.027
**At 2 years**					
LVM (g)	101.51	(89.64–105.06)	208.07	(126.14–284.31)	<0.001
LVMI (g/m^2^)	69.11	(67.40–70.49)	113.08	(85.29–143.59)	<0.001
LVIDd (mm)	41.0	(37.0–44.0)	48.0	(46.3–60.0)	<0.001
LVIDs (mm)	24.0	(18.0–26.0)	30.0	(27.0–47.0)	<0.001
LA (mm)	37.0	(24.0–39.0)	44.5	(40.9–50.0)	<0.001
RV (mm)	19.0	(18.0–25.0)	25.0	(23.5–26.3)	0.001
IVS (mm)	8.0	(7.0–12.0)	13.0	(11.8–16.0)	<0.001
LVPW (mm)	9.0	(6.0–9.0)	12.0	(9.0–14.0)	<0.001
EDV index (ml/m^2^)	34.68	(30.03–38.82)	64.92	(51.00–75.30)	<0.001
ESV index (ml/m^2^)	11.3	(8.13–17.61)	23.6	(10.7–35.5)	0.056
LA volume (ml)	32.1	(22.6–51.0)	68.4	(45.8–83.3)	<0.001
LA volume index (ml/m^2^)	22.3	(17.0–33.5)	42.1	(32.9–45.0)	0.002
EF (%)	70.9	(54.9–73.2)	68.1	(50.6–78.5)	0.840
Kt/V	2.34	(2.02–2.41)	1.81	(1.50–2.12)	<0.001
WCC	54.65	(46.17–80.14)	60.22	(48.34–65.50)	0.482

Change in Kt/V was calculated at Kt/V at 2 years – baseline Kt/V; positive changes were classified as improvements and negative changes as worsening. Values are medians (interquartile range). *BSA*, body surface area; *IVSd*, interventricular septum thickness (diastolic); *LVPWd*, left ventricular posterior wall thickness (diastolic); *LVIDd*, LV internal diameter (diastolic); *LVIDs*, LV internal diameter (systolic); *LVMI*, LV mass index; *LA*, left atrium; *RV*, right ventricle; *LVEDV*, LV end-diastolic volume; *LVESV*, LV end-systolic volume; *LVEF*, LV ejection fraction; *SDI*, systolic dyssynchrony index; *NT-proBNP*, N-terminal pro-B-type natriuretic peptide; WCC, weekly creatinine clearance.

**Table 3:** Change in dialysis adequacy indices change and SDI at peak DSE

**Group**	**SDI ≤1.1%**	**SDI >1.1%**
n	23	25
Kt/V		
Baseline	2.06 (2.00–2.08)	1.86 (1.79–2.26)
2 years	2.34 (2.04–2.38)	1.72 (1.50–2.09)
* P*	0.165	<0.001
WCC		
Baseline	61.10 (51.35–69.99)	68.99 (61.14–79.4)
2 years	54.65 (51.38–78.93)	60.22 (39.25–63.13)
* P*	0.68	<0.001

Values are medians (interquartile range). *P*-values were determined using the Wilcoxon two related samples test. *DSE*, dobutamine stress echocardiography; *SDI*, systolic dyssynchrony index; *WCC*, weekly creatinine clearance.

### Factors influencing dialysis adequacy at 2 years

Multivariate linear regression analysis revealed that log NT-proBNP, SDI at peak dobutamine dose, sex, and systolic wall stress were independently associated with Kt/V at 2 years (Table 4). Multiple linear regression analysis identified log NT-proBNP, SDI at peak dobutamine dose, age are independent predictors of weekly creatinine clearance at 2 years (Table 5).

**Table 4:** Multivariate regression analysis with Kt/V at 2 years as the dependent variable

**Predictors**	**Regression coefficient**	**Standard error**	**Standardized coefficient**	***P***
Intercept	3.199	0.184		0.000
Sex	−0.280	0.124	−0.355	0.030
Age	0.003	0.002	0.062	0.220
SDI at peak dose	−0.054	0.014	−0.295	0.001
logNT-proBNP	−0.154	0.044	−0.295	0.001
LVMI	0.001	0.001	0.228	0.237
SWS	−0.008	0.000	−0.785	0.000
R^2^=0.971				

*SDI*, systolic dyssynchrony index; *NT-proBNP*, N-terminal pro-B-type natriuretic peptide; *SDI*, systolic dyssynchrony index; *LVMI*, left ventricular mass index; *SWS*, systolic wall stress.

**Table 5:** Multivariate regression analysis with WCC at 2 years as the dependent variable

**Predictors**	**Regression coefficient**	**Standard error**	**Standardized coefficient**	***P***
Intercept	−86.124	17.761		0.000
Sex	−23.627	11.957	−0.780	0.055
Age	0.940	0.196	0.602	0.000
SDI at peak dose	−4.265	1.374	−0.606	0.004
logNT-proBNP	−0.154	0.044	−0.295	0.001
LVMI	−0.082	0.117	−0.333	0.488
SWS	−0.009	0.043	−0.023	0.827
R^2^=0.815				

*WCC*, weekly creatinine clearance; *SDI*, systolic dyssynchrony index; *NT-proBNP*, N-terminal pro-B-type natriuretic peptide; *SWS*, systolic wall stress; *LVMI*, left ventricular mass index.

### Factors influencing dynamic dyssynchrony

Multivariate linear regression analysis showed that log NT-proBNP and sex were independently associated with SDI at peak dobutamine dose (Table 6).

**Table 6:** Multivariate regression analysis with SDI at peak DSE as the dependent variable

**Predictors**	**Regression coefficient**	**Standard error**	**Standardized coefficient**	***P***
Intercept	5.881	3.337		0.086
Sex	−2.732	0.654	−0.688	0.000
Age	0.037	0.034	0.166	0.281
EF	−0.137	0.074	−0.622	0.071
logNT-proBNP	3.050	0.398	1.056	0.000
LVMI	−0.031	0.027	−0.411	0.251
EDV index	0.086	0.046	0.762	0.072
ESV index	−0.147	0.104	−0.740	0.165
SWS	−0.012	0.008	−0.214	0.134
R^2^=0.750				

*SDI*, systolic dyssynchrony index; *DSE*, dobutamine stress echocardiography; *EDV*, end-diastolic volume; *ESV*, end-systolic volume; *EF*, ejection fraction; *SWS*, systolic wall stress ; *NT-proBNP*, N-terminal pro-B-type natriuretic peptide.

### Intraobserver and interobserver variability

The interobserver variability for SDI was 6.3%, while intraobserver variability was 5.7%.

## Discussion

DSE is a useful tool for evaluating cardiovascular risk in ESRD patients who are potential candidates for renal transplantation [12]. It also provides a comprehensive assessment of changes in echocardiographic parameters during each stage by using novel 3D echocardiographic tools [13]. 3D DSE is now widely considered as an alternative to exercise testing to accurately and reproducibly assess dynamic LV dyssynchrony [14]. Here, we report novel results showing that CAPD patients have more obvious LV systolic dyssynchrony in response to dobutamine stress, despite normal LVEF and similar resting dyssynchrony index, compared with healthy individuals. The difference in stress-induced dyssynchrony between CAPD patients and healthy controls is probably caused by LV hypertrophy (LVH) in the former, as CAPD patients are more likely to develop LVH. LVH is associated with increased myocardial oxygen demand and abnormal coronary flow, and may result in subendocardial ischemia or regional dysfunction during stress [15]. Differences in volume status may also be responsible for the difference, as volume overload is more common in CAPD patients than in the normal population [16]. Additionally, previous reports revealed that volume status may modulate LV dyssynchrony [17,18]. Aljaroudi et al. reported that, among patients with ESRD, the prevalence of all-cause mortality is higher in patient with than in those without LV dyssynchrony [19]. A greater degree of dyssynchrony increased the 2-year mortality rate by 4–7-fold in patients free of high-risk features such as depressed LVEF, wide QRS, increased LV mass, or a large perfusion defect. Their study highlights the importance of cardiac dyssynchrony in terms of its detrimental effects on prognosis. We are eager to known whether its detrimental effects on prognosis are mediated by dialysis adequacy or cardiac structure.

An earlier study revealed that cardiac structure and function are influenced by uremic status and adequate dialysis therapy in pediatric CAPD patients; however, that was a cross-sectional study rather than a longitudinal study [20]. It is unknown whether cardiac function has an impact on long-term dialysis adequacy. So far, there are no data showing which echocardiographic parameters can predict long-term dialysis adequacy. In our longitudinal study, we clearly showed that dynamic dyssynchrony (SDI at peak DSE) and logNT-proBNP were significantly associated with indices of dialysis adequacy at 2 years (Kt/V and WCC; as shown in Tables 4 and 5). In patients with low dynamic SDI, the dialysis adequacy indices did not change significantly. However, in patients with high dynamic SDI, the dialysis adequacy indices tended to deteriorate over time, as shown in Table 3. A possible explanation for the dynamic dyssynchrony-induced deterioration of dialysis adequacy is as following. Stress-induced dyssynchrony may decrease the forward stroke volume, decreasing blood flow to the peritoneal capillaries responsible for solute clearance. This was observed in heart failure subjects with preserved ejection fraction [21]. The clinical scenario is very similar in CAPD patients who often have LV hypertrophy but preserved ejection fraction. Because we only included non-ischemic patients in this study, we did not observe a significant difference in troponin I concentrations between patients with high or low SDI. Adequate solute and fluid removal is the cornerstone of dialysis therapy and both are important targets of dialysis adequacy. Fluid overload is often seen in PD patients, and may contribute to high cardiac mortality [22,23]. However, it is difficult to determine the volume status in PD patients using routine clinical methods [24]. NT-proBNP is synthesized in the LV in response to greater ventricular work, and is a marker of myocardial damage and fluid overload [6]. As shown in Table 6, log NT-proBNP was the most important predictor of cardiac dyssynchrony during the stress test, even after adjusting for other traditional parameters. Log NT-proBNP was also independently associated with PD adequacy, as shown in Tables 4 and 5. Therefore, elevated NT-proBNP levels may reflect fluid overload in CAPD patients, and is easy to measure in clinical settings. As indicated in Table 2, CAPD subset with a greater improvement in Kt/V change at 2 years showed less systolic LV wall stress as compared with patients with poorer improvements in Kt/V. Altered myocardial stress can result in the production of cytokines and reactive oxygen species, which can stimulate myocyte apoptosis [25] and extracellular matrix disruption [26], important triggers for destructive LV dilatation and remodeling [27]. Progressive LV dilatation and changes in chamber shape (i.e., increasing sphericity) can further stretch myocytes and upregulate the expression of stretch response proteins, which can induce pathologic cardiomyocyte hypertrophy [28]. In the current study, we found that patients with improvements in Kt/V had favorable echocardiographic parameters, including a lower LVMI, a smaller LV chamber, and a thinner LV wall, as compared with patients with a worsening in Kt/V. Taken together, our results suggest that higher dynamic LV dyssynchrony could predict lower Kt/V and unfavorable echocardiographic outcomes 2 years later.

We found that female CAPD patients had more pronounced dynamic LV dyssynchrony than males. This finding is consistent with the findings reported by Kuznetsova et al [29],. who reported that, in a study population with LV diastolic dysfunction, females had greater dyssynchrony compared with males. The differences in susceptibility between sexes could be related to sex hormones, such as estrogen [30].

Some limitations should be discussed. For example, the study sample size was relatively small and we only examined systolic dyssynchrony; regression analysis cannot address causality, only associations between two factors; and other unrecorded/untested parameters may influence the results.

## Conclusions

Dynamic systolic dyssynchrony was strongly associated with future dialysis adequacy in CAPD patients. Log NT-proBNP was the important predictor of dynamic dyssynchrony. Our study confirmed the concept that cardiac dysfunction has an impact on dialysis adequacy. Future studies should evaluate stress-related diastolic dyssynchrony, and the associations in different study populations, such as patients with stage III–V CKD.

## Competing interests

All authors declared that they have no competing interest.

## Authors’ contributions

HCH & CCC contributed in study design and data collection and all authors contributed in editing and drafting the manuscript. All authors read and approved the final manuscript.

## Pre-publication history

The pre-publication history for this paper can be accessed here:

http://www.biomedcentral.com/1471-2369/14/68/prepub
